# PIK3CA and KRAS mutations in cell free circulating DNA are useful markers for monitoring ovarian clear cell carcinoma

**DOI:** 10.18632/oncotarget.24555

**Published:** 2018-02-22

**Authors:** Asuka Morikawa, Tomoatsu Hayashi, Naomi Shimizu, Mana Kobayashi, Kenzui Taniue, Akiko Takahashi, Kota Tachibana, Misato Saito, Ayako Kawabata, Yasushi Iida, Kazu Ueda, Motoaki Saito, Nozomu Yanaihara, Hiroshi Tanabe, Kyosuke Yamada, Hirokuni Takano, Osamu Nureki, Aikou Okamoto, Tetsu Akiyama

**Affiliations:** ^1^ Laboratory of Molecular and Genetic Information, Institute of Molecular and Cellular Biosciences, The University of Tokyo, Tokyo, Japan; ^2^ Department of Obstetrics and Gynecology, Jikei University School of Medicine, Tokyo, Japan; ^3^ Department of Obstetrics and Gynecology, Jikei University, Kashiwa Hospital, Chiba, Japan; ^4^ Department of Biological Sciences, Graduate School of Science, The University of Tokyo, Tokyo, Japan

**Keywords:** OCCC, cfDNA, PIK3CA, KRAS, digital PCR

## Abstract

Ovarian clear cell carcinoma (OCCC) exhibits distinct phenotypes, such as resistance to chemotherapy, poor prognosis and an association with endometriosis. Biomarkers and imaging techniques currently in use are not sufficient for reliable diagnosis of this tumor or prediction of therapeutic response. It has recently been reported that analysis of somatic mutations in cell-free circulating DNA (cfDNA) released from tumor tissues can be useful for tumor diagnosis. In the present study, we attempted to detect mutations in PIK3CA and KRAS in cfDNA from OCCC patients using droplet digital PCR (ddPCR). Here we show that we were able to specifically detect PIK3CA-H1047R and KRAS-G12D in cfDNA from OCCC patients and monitor their response to therapy. Furthermore, we found that by cleaving wild-type PIK3CA using the CRISPR/Cas9 system, we were able to improve the sensitivity of the ddPCR method and detect cfDNA harboring PIK3CA-H1047R. Our results suggest that detection of mutations in cfDNA by ddPCR would be useful for the diagnosis of OCCC, and for predicting its recurrence.

## INTRODUCTION

Tumor tissues release cell-free circulating DNA (cfDNA) into the blood, and some of this DNA contains somatic mutations diagnostic of the cancer [1–3]. cfDNA is fragmented to an average length of 140 ∼ 170 bp and is present in only a few thousand amplifiable copies per milliliter of blood. Recent progress in polymerase chain reaction (PCR) and next-generation sequencing (NGS) technology has made it possible to detect somatic mutations characteristic of tumor tissues by measuring small amounts of cfDNA, e.g. PIK3CA-H1047R, -E545K and -E542K in breast cancer [4], BRAF-V600E and -V600K and -NRAS-Q61H in melanoma [5] and EGFR-T790M in non-small cell lung cancer [6]. Furthermore, accumulating evidence suggests that cfDNA is also useful for monitoring the response to therapy [7–10].

Ovarian clear cell carcinoma (OCCC) is one of the four major histological subgroups of epithelial ovarian cancer: i.e. serous, clear cell, endometrioid and mucinous. OCCC has distinct characteristics, such as resistance to chemotherapy, poor prognosis, an association with endometriosis, a higher incidence of thrombosis as a complication and a higher incidence among Japanese [11–13]. OCCC develops as a result of mutations in various tumor suppressors and oncogenes, including ARID1A (at-rich interaction domain-containing protein 1A), PIK3CA (phosphatidylinositol 3-kinase catalytic subunit), KRAS and TP53 [14, 15]. In the present study, we attempted to detect mutations in PIK3CA and KRAS in cfDNA from OCCC patients using droplet digital PCR (ddPCR). We show that this method is useful for monitoring the therapeutic response of OCCC.

## RESULTS AND DISCUSSION

**Figure 1:**
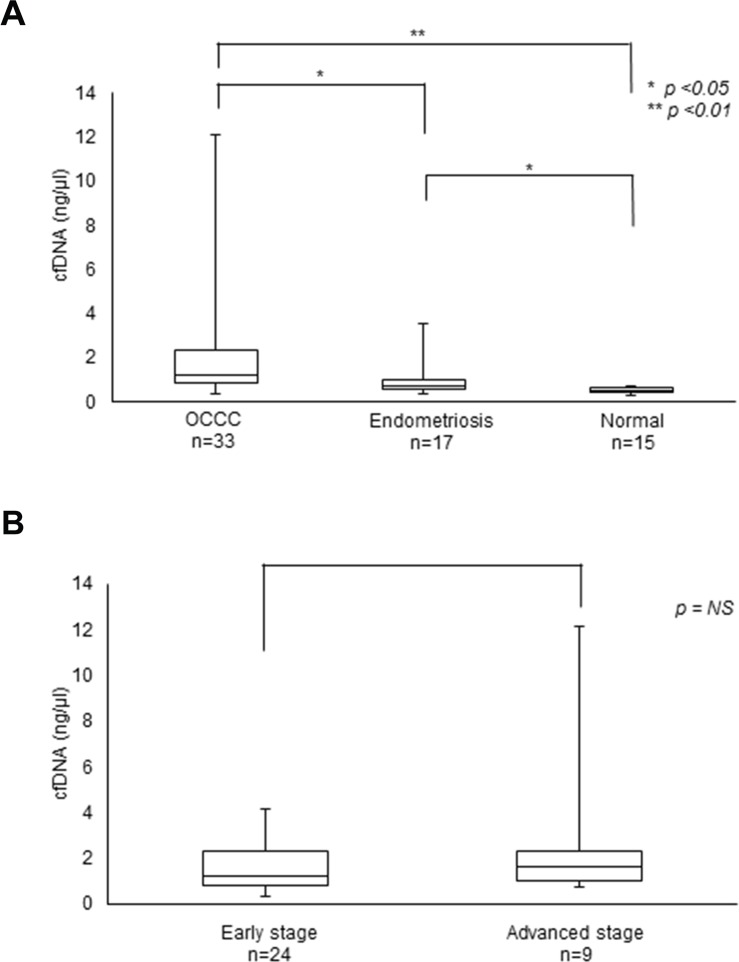
Levels of cfDNA in OCCC patients **(A)** cfDNA levels in OCCC and endometriosis patients. **(B)** cfDNA levels at the early and advanced stages of OCCC.

We first compared the average amounts of cfDNA and CA125 (carbohydrate antigen 125), a biomarker for some cancers, including OCCC, in blood samples collected from 33 OCCC patients before surgery [partial median age = 53.5 years old (38 – 71 years old)]. Consistent with previous reports [16], OCCC patients and patients with endometriosis had significantly higher levels of cfDNA than did healthy people (Table 1, Figure 1). On the other hand, we found no obvious differences in the concentration of cfDNA between early stage and advanced stage cancers.

**Table 1:** Levels of cfDNA and CA125 in OCCC patients

	n	cfDNA^*^ (median; ng/μl)	Range	CA125 (median; U/ml)	Range
**Clear Cell Carcinoma**					
**Stage**					
**I**	21	1.23	(0.35 - 4.13)	32.5	(6 - 979)
**II**	3	0.91	(0.51 - 2)	481.0	(239 - 723)
**III**	8	1.77	(0.73 - 12.4)	539.0	(21 - 2,190)
**IV**	1	0.84		56.0	
**Total**	33	1.23	(0.51 - 12.4)	57.0	(6 - 2,190)
**Normal**	15	0.54	(0.29 - 0.7)		
**Endometriosis**	17	0.75	(0.36 - 3.56)		

^*^cfDNA : cell free DNA per 1 ml plasma.

PIK3CA is frequently mutated in OCCC (∼ 50%) [15]. Furthermore, PIK3CA-H1047R is a hotspot and can be detected in most of the patients that have PIK3CA mutation using only one set of primers. KRAS-G12D can be detected in ∼4% of patients that possess a KRAS mutation [17], also using one set of primers. By contrast, although ARID1A is the most frequently mutated gene in OCCC (more than ∼ 60%), these mutations occur throughout the gene and require numerous primer sets in order to detect. We therefore attempted to detect PIK3CA-H1047R and KRAS-G12D in cfDNA from OCCC patients by droplet digital PCR (ddPCR). ddPCR analysis specifically detected PIK3CA-H1047R and KRAS-G12D in both cfDNA and tumor tissues, but not in normal tissues or leukocytes from patients OCCC13 and OCCC14, respectively (Table 2). When we analyzed tumor tissues and cfDNA from 29 patients, we detected PIK3CA-H1047R in tumor tissues from 5 patients and in cfDNA from 2 out of the 5 patients (Table 3). We also detected KRAS-G12D in tumor tissues from 3 patients and cfDNA from 1 out of the 3 patients.

**Table 2:** Specificity of ddPCR assays

PIK3CA H1047R	Copies/ml	
	Mutant	Wild-type
OCCC13 Tumor tissue	499	46,400
OCCC13 Normal tissue	0	38,000
OCCC13 cfDNA	165	63,300
Wild-type tissue (n=10)	0	471,600
Normal cfDNA (n=3)	0	108,800
Non-Template Control	0	0
**KRAS G12D**	**Copies/ml**	
	**Mutant**	**Wild-type**
OCCC14 Tumor tissue	13,300	21,500
OCCC14 Normal tissue	0	52,800
OCCC14 cfDNA	285	47,700
Wild-type tissue (n=10)	0	532,200
Normal cfDNA (n=3)	0	88,250
Non-Template Control	0	0

**Table 3:** Detection of PIK3CA-H1047R and KRAS-G12D in cfDNA

Sample number (n=29)	PIK3CA-H1047R			KRAS-G12D	
	Stage	Tumor	cfDNA (copies/ml)	Tumor	cfDNA (copies/ml)
OCCC1	III				
OCCC2	I				
OCCC3	I				
OCCC4	I	•	0		
OCCC5	I				
OCCC6	I				
OCCC8	III				
OCCC10	III				
OCCC11	II				
OCCC12	I	•	0		
OCCC13	IV	•	165		
OCCC14	III			•	285
OCCC15	III				
OCCC16	I				
OCCC17	I	•	275		
OCCC18	I				
OCCC19	I				
OCCC21	I			•	0
OCCC22	I				
OCCC24	I				
OCCC25	II	•	0^*^		
OCCC26	I				
OCCC27	I			•	0
OCCC31	I				
OCCC33	I				
OCCC34	III				
OCCC35	I				
OCCC36	I				
OCCC37	II				

^*^Although cfDNA was 0 at the initial diagnosis, cfDNA was detected during the course of disease.

**Figure 2:**
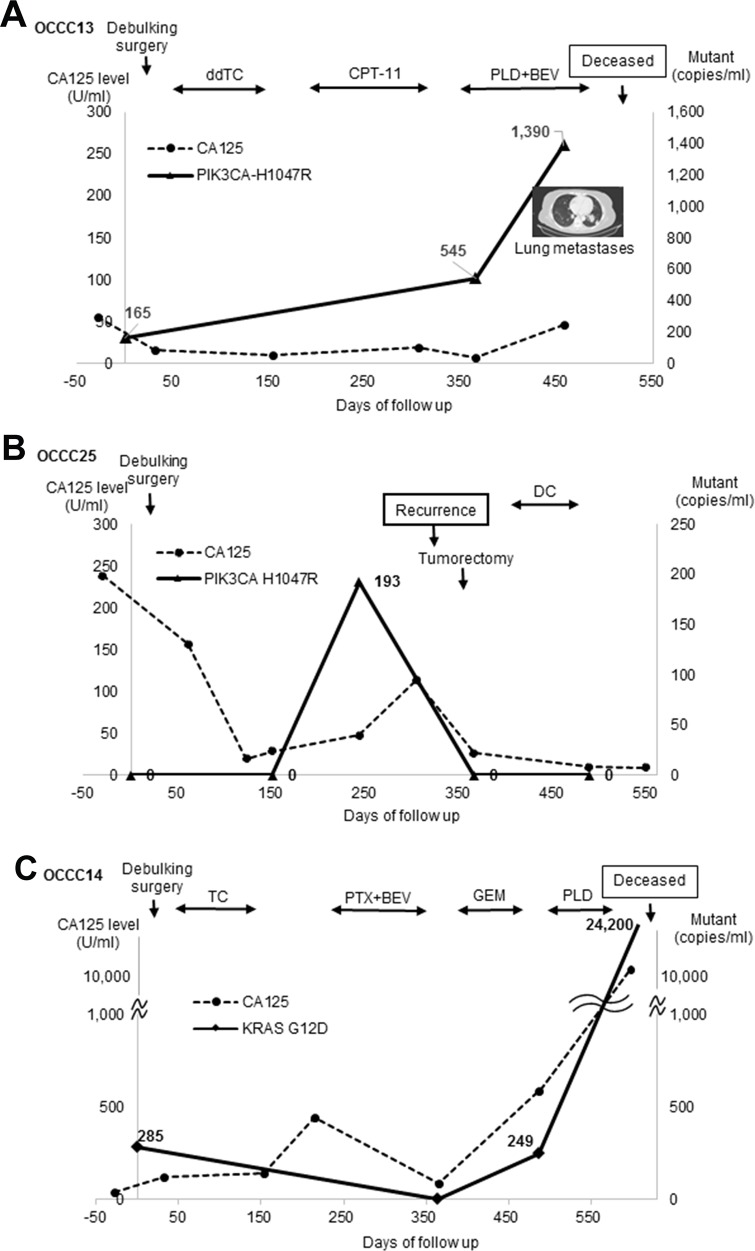
cfDNA mutations and disease development **(A)** Patient OCCC13. ddTC, dose dense weekly paclitaxel and carboplatin; BEV, bevacizumab + CPT-11 + irinotecan; PLD, doxorubicin. **(B)** Patient OCCC25. DC, docetaxel + carboplatin. **(C)** Patient OCCC14. PTX, paclitaxel; GEM, gemcitabine.

We next assessed PIK3CA-H1047R and/or KRAS-G12D levels in cfDNA from three patients during the course of the disease. Patient OCCC13, who was diagnosed with stage IV disease and had multiple lung metastasis, received three-step chemotherapy after debulking surgery (Figure 2A). PIK3CA-H1047R was detected at the time of surgery and dramatically increased after one year, at which time OCCC had further metastasized to the bone. By contrast, the level of CA125 increased less dramatically than that of PIK3CA-H1047R. In the case of Patient OCCC25 (stage II), PIK3CA-H1047R level was drastically increased 250 days before debulking surgery (Figure 2B). This increase in PIK3CA-H1047R levels was followed by the recurrence of disease and an increase in CA125 levels. After tumorectomy, the levels of PIK3CA-H1047R and CA125 were reduced to undetectable levels. In the case of OCCC14 (stageIII), KRAS-G12D levels were decreased after surgery and adjuvant chemotherapy, but dramatically increased before death (Figure 2C).

**Figure 3:**
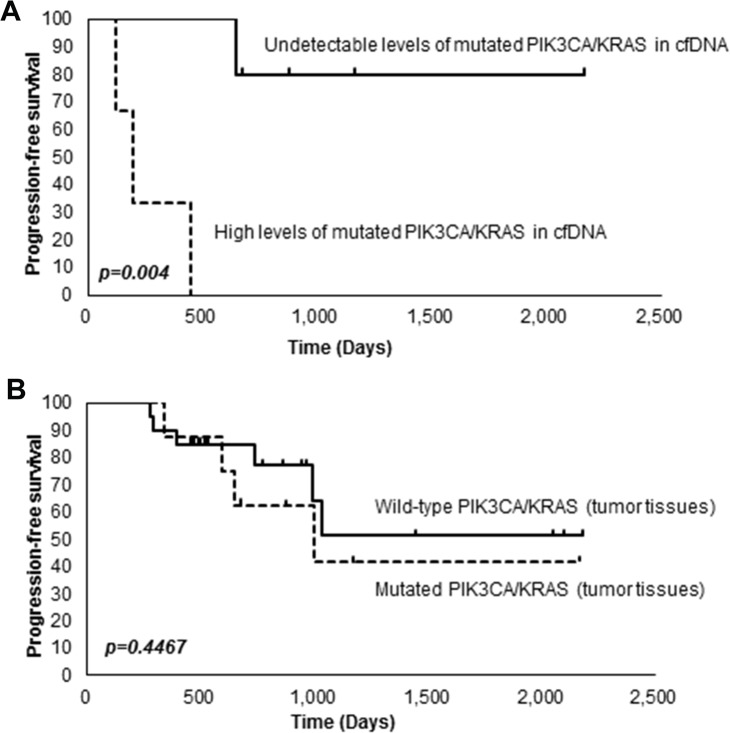
Kaplan-Meier analysis of OCCC patients **(A)** Association between the levels of PIK3CA-H1047R or KRAS-G12D in cfDNA and survival. **(B)** Association between mutations in PIK3CA or KRAS in tumor tissues and survival.

We next investigated the relationship between mutations in PIK3CA or KRAS and survival. A Kaplan-Meier survival analysis revealed that patients with high levels of PIK3CA-H1047R or KRAS-G12D in cfDNA (≧ 1 copy/ml) had significantly shorter progression-free survival (PFS) than patients having undetectable levels of PIK3CA-H1047R or KRAS-G12D in cfDNA (= 0 copies/ml) (*P* < 0.004) (Figure 3A). On the other hand, we did not observe any significant association between mutations in PIK3CA or KRAS in tumor tissues and survival (Figure 3B).

**Figure 4:**
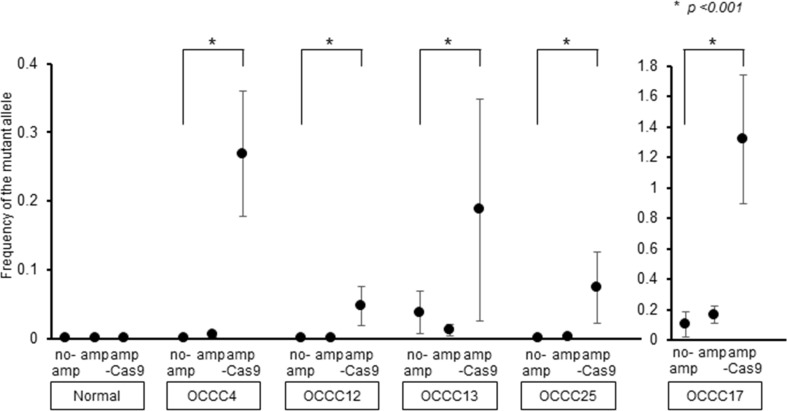
Improvement of the sensitivity of PIK3CA-H1047R detection cfDNA fragments harboring PIK3CA (no am) were amplified by ddPCR (am) and then fragments encoding wild-type PIK3CA were cleaved using the CRISPR/Cas9 system (am-Cas9). PIK3CA-H1047R was detected at each step by ddPCR. All data represent mean ± SEM (n = 3). ^*^P < 0.05 (*t* test).

To improve the sensitivity of ddPCR for detecting cfDNA harboring PIK3CA-H1047R, we used the CRISPR/Cas9 system to specifically cleave cfDNA fragments encoding wild-type PIK3CA. cfDNA harboring PIK3CA was amplified and incubated with Cas9 and a single guide RNA (sgRNA) specific for wild-type PIK3CA. Analysis of the reaction products by ddPCR revealed that cleavage of wild-type PIK3CA resulted in the increased detection of PIK3CA-H1047R in cfDNA from patients OCCC4, 12, 17 and 25 (Figure 4). Thus, removal of wild-type PIK3CA by CRISPR/Cas9 may lead to enhanced detection of PIK3CA-H1047R by ddPCR.

Although PIK3CA mutations have been detected in cfDNA from patients with breast or colorectal cancers [4, 18], there have been no reports investigating ovarian cancer. In the present study, we successfully detected PIK3CA-H1047R and KRAS-G12D in cfDNA from OCCC patients using ddPCR, and were able to monitor the response to therapy and to detect cancer relapse. Of particular interest is the fact that the increase in PIK3CA-H1047R levels preceded recurrence and an increase in CA125 levels in patient OCCC25. In addition, PIK3CA-H1047R levels increased more significantly than CA125 levels in patients OCCC13 and OCCC25. These results suggest that detection of PIK3CA-H1047R would be useful for the diagnosis of OCCC and for predicting its recurrence. Since PIK3CA mutation is an early event in the development of endometriosis-associated ovarian carcinomas [19, 20], detection of PIK3CA-H1047R in cfDNA from endometriosis patients may also be useful for early diagnosis of OCCC. We also found that patients with high levels of PIK3CA-H1047R or KRAS-G12D in cfDNA had significantly shorter PFS than patients having undetectable levels of PIK3CA-H1047R or KRAS-G12D in cfDNA. On the other hand, as reported previously [21, 22] we did not observe any significant relationship between mutations in PIK3CA or KRAS in tumor tissues and survival. Finally, we succeeded in improving the sensitivity of this method by using the CRSPR/Cas9 system to cleave wild-type PIK3CA prior to ddPCR. We imagine that this method can also be used to detect other types of cancers.

## MATERIALS AND METHODS

### Patients

Plasma samples and tumor tissues were obtained from 33 patients diagnosed with OCCC at the Jikei University Hospital [23]. Plasma samples were collected just before debulking surgery. Plasma samples from 18 patients (OCCC2∼6, OCCC12∼19, OCCC21 and OCCC24∼27) were obtained every 6 months after surgery. All the patients signed an informed consent form, approved by the ethics committee of The Jikei University School of Medicine.

### DNA extraction

Plasma was collected from EDTA tubes by centrifugation at 3,500 × g for 10 min or from Cell-Free DNA BCT tubes (Streck) by centrifugation at 1,600 × g for 10 min at 4°C. The supernatant was centrifuged at 16,000 g for 10 min. Plasma was stored at −80°C until use. DNA was extracted from plasma with the QIAamp Circulating Nucleic Acid Kit (Qiagen) according to the manufacturer's instructions and quantitated using the Qubit 2.0 high sensitivity DNA kit (ThermoFisher). DNA was isolated from tumor and normal tissues using the AllPrep DNA/RNA Mini Kit (Qiagen).

### Droplet-based digital PCR (ddPCR)

KRAS-G12D was detected with ddPCR on the QX100TM droplet digital PCR system (Bio-Rad) using Bio-Rad's PrimePCR mutation and wild-type assays. For detection of PIK3CA-H1047R, a dual-labeled locked nucleic acid (LNA) probe strategy was used (mutant probe sequence, 5’- FAM C+CATG+A+C+GTGCA-Iowa black FQ-3′; wild-type probe sequence, 5’- HEX C+CATG+A+T+GTG+CA - Iowa black FQ-3′). All probes corresponding to mutant or wild-type alleles were labelled with either 6-FAM or HEX fluorophores. Reaction mixtures (20 μl) containing 2.5 ng or 5 ng of digested sample DNA, ddPCR Supermix for probes (Bio-Rad), 1,000 nM of each primer and 250 nM of each probe were loaded into the QX100 Droplet Generator. The samples were amplified on a conventional Bio-Rad T100 Thermal Cycler (95°C for 10 min, followed by 40 cycles of 95°C for 30 sec and 60°C (PIK3CA-H1047R) or 55°C (KRAS-G12D) for 60 sec, with a final elongation step of 98°C for 10 min). The plate, containing the droplet amplicons, was subsequently loaded into the QX100 Droplet Reader (Bio-Rad).

### Pre-amplification using nested PCR

A 107 bp fragment of PIK3CA containing the codon for amino acid residue 1047 was amplified from cfDNA by ddPCR. cfDNA (2.5 ng) from patients was amplified in a 20-μl reaction solution containing 10x KOD buffer (Toyobo), 0.2U KOD plus (Toyobo), 2x Droplet supermix (Bio-Rad) and 10 μM primers (nested primers: Fw 5’-ACTGAGCAAGAGGCTTTGGA-3’ and Rv 5’-GCATGCTGTTTAATTGTGTGG-3’). The entire reaction mixture was loaded into a droplet generator (Bio-Rad). PCR pre-amplification was carried out on a T100 thermal cycler (Bio-Rad) using a thermal profile beginning with 95°C for 10 min, followed by 9 cycles of 95°C for 30 sec and 60°C for 60 sec, and finally 1 cycle of 98°C for 10 min. Droplets of PCR products were disrupted, 200 μl chloroform and 80 μl TE Buffer were added, and the sample was vortexed for 1 min, then centrifuged at 10,000 × g for 10 min. The supernatants were removed and purified using MinElute (Qiagen) following the manufacturer's instructions. One microliter of the first PCR product was re-amplified by ddPCR using the LNA probe and the primer for PIK3CA-H1047R and was detected as described above.

### Cleavage of wild-type PIK3CA cfDNA *in vitro*

To enhance the sensitivity of ddPCR for detecting cfDNA harboring PIK3CA-H1047R, cfDNA fragments encoding wild-type PIK3CA were specifically cleaved *in vitro* using the Guide-it Complete sgRNA Screening System (Takara Bio) following the manufacturer's instructions with slight modifications. Briefly, cfDNA that had been PCR amplified using nested primers as described above was incubated with purified Cas9 protein [24] and *in vitro* transcribed sgRNA specific for wild-type PIK3CA (target sequence: 5’-caaatgaatgatgcacatca-3’) in Cas9 nuclease reaction buffer (Takara Bio) at 37°C for 1 hr. Reactions were stopped by incubation at 70°C for 10 min. The products cleaved by Cas9 were purified using MinElute (Qiagen) and analyzed by ddPCR.

### Statistical analysis

All statistical analyses were performed using Statview software (version 5.0.1.; SAS Institute Inc., Cary, NC). Survival rates were calculated by the Kaplan-Meier method. The differences in survival were tested using the log-rank test. *T* tests were used to compare normally distributed continuous data.
